# Cross-sectional examination of tobacco point-of-sale marketing practices by location and type of retail environment in the District of Columbia

**DOI:** 10.18332/tpc/211431

**Published:** 2025-10-31

**Authors:** Melissa Hawkins, Charis Edwards, Bright Amenyo, Jackie White, Karima Boumatar, Paola Koki Ndombo, Carrie Dahlquist, Anastasia Snelling

**Affiliations:** 1 Department of Health Studies, College of Arts and Sciences, American University, Washington DC, United States; 2 DC Department of Health, Tobacco Control Program, Washington DC, United States

**Keywords:** tobacco, point-of-sale marketing, advertising, tobacco retail, price promotion, tobacco control

## Abstract

**INTRODUCTION:**

Despite national and state policy interventions and public health efforts in tobacco control, tobacco use remains the leading cause of preventable death, disease, and disability in the United States. Point-of-sale marketing in the retail environment significantly contributes to tobacco initiation and use among youth and adults, particularly in communities with lower income. The aim of the study was to assess the retail landscape and marketing practices for tobacco products by location and retail store environment.

**METHODS:**

This cross-sectional study examined the retail landscape and marketing practices of tobacco products among a random sample of licensed retailers in the District of Columbia (DC) (n=264) from June 2024 to April 2025, including product availability, promotion, price, and placement. Predictors of marketing practices were evaluated by geographical location and store type for flavored and non-flavored products including cigarettes, cigarillos, cigars, chew/snuff/loose tobacco, e-cigarettes, and hookah. Data were collected using an adapted version of the Standardized Tobacco Assessment for Retail Settings (STARS) instrument.

**RESULTS:**

The most available products were cigarillos (80%, n=215), cigarettes (78%, n=206), and chew/snuff/loose tobacco (67%, n=176). Convenience stores were more likely to have price promotions [χ^2^(2, N=264)=13.4, p<0.01], tobacco products placed <12 inches of toys, candy, or gum [χ^2^(2, N=264)=16, p<0.001], and tobacco products advertised on the store exterior [χ^2^(2, N=264)=24.9, p<0.001] compared to all other store types. Lower income communities also had a higher frequency of price promotions [χ^2^(7, N=264)=34.99, p<0.001], products placed within 12 inches of toys, candy, or gum, [χ^2^(7, N=264)=41.28, p<0.001] and tobacco products advertised on the store exterior. We found significantly more types of marketing in lower income communities, particularly for cigarillos, which were disproportionately available, marketed, and lower priced.

**CONCLUSIONS:**

There are differences in the way tobacco products are marketed and promoted across DC based on store type and location. Youth and adults in communities with lower income are at increased risk of being exposed to tobacco products and marketing. Additional studies examining restrictions on point-of-sale marketing practices, particularly price promotions, are important to complement regulatory interventions and policies.

## INTRODUCTION

Tobacco products are among the most marketed consumer products in the United States (US). In 2022, cigarette manufacturers spent over $8 billion on advertising and promotional activities in retail environments, with >85% of the spending dedicated to point-of-sale (POS) marketing efforts^1^. POS marketing strategies include price discounts, product placement in highly visible locations, promotional activities, incentives for retailers to increase in-store marketing, and exterior and interior advertisements^2^. POS strategies in the tobacco retail environment often focus on the four Ps of marketing: product, price, placement, and promotion^3^. However, these strategies are highly localized with greater concentrations of POS tobacco marketing in lower income communities^4-6^.

Tobacco product POS marketing has long been established as a significant environmental risk factor for tobacco initiation and use^7^. Previous studies demonstrate that tobacco marketing strategies have a direct impact on smoking initiation^8^, prevalence^9^, and tobacco purchases^10^, particularly among adolescents^11,12^. Community health is impacted by the tobacco retail environment^11-13^ and greater exposure to POS marketing may contribute to tobacco-related health disparities experienced by racial/ethnic minority and low-income groups. For example, advertising of menthol cigarettes and flavored cigarillos is more frequent in low-income communities, leading to higher rates of tobacco initiation^14^.

Although national and state policy interventions and public health efforts have contributed to significant progress in tobacco prevention and control, tobacco use remains the leading cause of preventable death, disease and disability in the US^15^. Smoking prevalence rates are inversely associated with household incomes^2^. Since 2009, the US Food and Drug Administration (FDA) has had the authority to regulate tobacco product marketing and promotion efforts through enactment of the Tobacco Control Act^16^. Recently, states in the US and many other countries have implemented regulations on flavored tobacco products^17^. State and local POS tobacco control typically prioritize limited size and placement of ads, prohibit the sale of tobacco products in stores with a pharmacy, fines for non-compliance, and tobacco-use warning signs, as mandated by the 2023 Racketeer Influenced and Corrupt Organizations Act (RICO)^18^.

Washington, D.C. (DC) comprises eight Wards and 702250 residents^19^, with the largest population in Ward 7 (90898 residents) and the smallest population in Ward 3 (78404 residents^20^. There are significant economic disparities observed by Ward and race. For example, Wards 2 and 3 have the highest percentage of White residents and the lowest percentage of Black/African American residents, whereas Wards 7 and 8 have the highest percentage of Black/African American residents and the lowest percentage of White residents^20,21^. Similarly, the median household income in Wards 2 and 3 is $132127 compared to $47706 in Wards 7 and 8^20^. Smoking prevalence varies markedly by Ward, with highest rates in Wards with the lowest income communities, reflecting underlying socioeconomic and racial disparities^20^. Smoking prevalence (lifetime tobacco use, over >100 cigarettes) among DC residents is approximately 10.6%^22^. This varies based on Ward with the highest smoking prevalence in Wards 7 and 8 at approximately 18.7%. The 2023 Behavioral Risk Factor Surveillance System (BRFSS) data do not provide smoking prevalence rates for Wards 2 and 3^22^. There are also racial disparities in smoking-associated health conditions with higher rates of cardiovascular disease, hypertension, diabetes, asthma, and cancer seen among Black residents^20^. Recent studies conducted in DC found higher proportions of flavored tobacco product advertising in predominantly Black neighborhoods compared to other neighborhoods^6,13^.

In DC, retailers and wholesalers are required to obtain a license to sell tobacco and e-cigarette products. According to the DC Department of Licensing and Consumer Protection, convenience stores account for the most common type of retailer licensed to sell tobacco products^23^. The Cigarette Tax Act was enacted in 2018 in DC^24^, and is among the highest cigarette tax rates ($4.50 per pack) in the US. The 2021 Flavored Tobacco Prohibition Act (DC Law 24-25) prohibits the sale of all flavored tobacco, including menthol cigarettes and e-cigarettes, in DC^25^. It also prohibits the sale of all electronic smoking devices within a quarter mile of any middle or high school in DC^25^.

This study assessed the retail landscape and marketing practices for tobacco products in DC by location and retail store environment, adjusting for demographic confounding factors. The goal of this cross-sectional study was to describe socioeconomic and geographical disparities in tobacco marketing practices to inform policy recommendations. We hypothesize that tobacco retail stores in lower resourced communities will utilize more marketing strategies than stores in higher resourced communities.

## METHODS

The DC Department of Health (DC Health) partnered with a local university from June 2024 to April 2025 to conduct this cross-sectional study. The study objective was to assess the availability, promotion, price, and placement of tobacco products in a representative sample of tobacco retailers across DC using an adapted version of the Standardized Tobacco Assessment for Retail Settings (STARS) instrument^26^. The validated STARS tool was adapted to evaluate marketing practices (see the full instrument in the Supplementary file) according to DC tobacco policies and pilot tested in September 2024 (n=12). The modified 18-item STAR survey includes the availability of tobacco products and their flavors, prices of specific products (Marlboro Red cigarettes, Newport Menthol cigarettes, and Black and Mild cigarillos, if sold), indoor and outdoor promotions, and the placement of tobacco products within the store. Modifications included adding example photos of tobacco products for data collection, adding flavored products, and adding an open-ended notes section for additional observations. Marketing practices were evaluated for flavored tobacco products (currently prohibited in DC)^25^ and non-flavored products including cigarettes, cigarillos, cigars, chew/snuff/loose tobacco, e-cigarettes, and hookah.

### Study population

The DC Department of Licensing and Consumer Protection (DLCP)^23^ provided a list of all DC businesses holding retail tobacco licenses as of June 2024 (n=835). Research assistants verified the operating status for all tobacco retail stores. Hotels, bars, and restaurants were excluded due to the structure of the sale of tobacco products as well as the hours of operation. A random sample was selected from the 611 eligible tobacco retailers. Convenience stores represent the largest proportion of tobacco retailers in DC^23^ and were oversampled. Trained research assistants collected data from the tobacco retail stores.

### Measures

*Store type*

Tobacco retail store categories were defined as: convenience store/corner store/deli (with or without gas); drug store/pharmacy; liquor store; grocery store/supermarket; and tobacco shop. Store type was categorized as convenience store and non-convenience store in the final analysis.

*Geographical location*

The geographical location was described by DC Ward, ranging from 1 to 8. In the final analysis, Ward 3 was the reference category with the highest median income^20^ and lowest smoking prevalence^22^ compared to other Wards.

*Product availability*

Data collectors assessed if the following products were available: cigarillos/little cigars; large cigars; chew, moist or dry snuff, dip or snus; e-cigarettes; cigarettes; and hookah. Products were also classified based on flavor (menthol/mint), other flavored (non-menthol), and non-flavored.

*Promotion*

Promotions were assessed outside and inside the retail environment, including products advertised within 3 feet of the floor inside the store. Price promotions by product (yes/no) and flavor (menthol, other flavored, non-flavored) were assessed as well as specific cigarillo/little cigar price promotions (singles sold, advertisements for less than $1). All items were dichotomous (yes/no).

*Price*

The price for a single pack of Marlboro Red cigarettes, Newport Menthol cigarettes, and Black and Mild cigarillos was recorded, if available. The lowest price per pack (in dollars) was recorded in the survey. Mean price by product was reported in the final analysis.

*Placement*

Three items were assessed for placement strategies of visible products including products sold within 12 inches of toys, candy, gum, slushy/soda machines, or ice cream; products sold within 3 feet of floor; and visible self-service display or vending machine for tobacco products.

*Smoking and income*

Lifetime smoking prevalence (‘Have you smoked at least 100 cigarettes (5 packs) in your entire life?’) and income (% of DC residents living below the poverty level) as of 2024 were included through secondary data sources, the BRFSS and DC Office of Planning, Data Analysis and Visualization Unit^22^.

*Covariates*

Regulatory compliance was documented by signs that displayed visible health warnings, signs stating the minimum age to purchase products, and RICO corrective statements. Stores participating in USDA Supplemental Nutrition Assistance Program (SNAP) were also identified. These variables were examined as potential confounders of the associations between tobacco store type, geographical location, and marketing strategies.

### Procedures

Data were collected using a Qualtrics online survey tool. The STARS instrument includes data training materials and a pocket guide with visuals for data collection in the field. Research assistants were trained in a group setting to practice data collection according to the study protocol under a variety of scenarios. Exclusion criteria included hotels, bars, and retailers that were not open on weekdays between 9 a.m. and 3 p.m. The sample was selected by both store type and Ward. The overall proportion of stores to be sampled was 41%. We applied an average of roughly 41% across store type based on how much variability we assumed there will be within each store type category. Further, we assumed highest variability across convenience stores, and sampled higher proportions within this store type category. Of the 611 eligible stores, we randomly selected 468 stores and visited 410 stores within the data collection study time period. An interactive map of the stores in the sample was developed to monitor data collection and to minimize travel time for data collection. Research assistants collected data in person in pairs on weekdays, surveying an average of 5–15 stores per day. Verbal assent was attained from the clerk and/or manager on duty prior to data collection activities.

### Data analysis

Each store was assigned a unique, three-digit identifier (UID). Descriptive statistics (mean and standard deviation, frequency and percentage) were analyzed to describe each survey item and to summarize sample characteristics. Data were entered, cleaned, and checked for accuracy by trained research assistants. The analysis was examined by geographical location (Ward) and tobacco retail store type. Differences in marketing strategies between geography and store types were examined using t-test or ANOVA for continuous variables and chi-squared or Fisher’s exact test for categorical variables. Stepwise backward multiple linear and logistic regression were performed to examine predictors of marketing strategies. Regression models were adjusted for confounding covariates. Adjusted and unadjusted regression coefficients (β), standard errors (SE), and 95% confidence intervals (CI) are reported for the four final models (product, price, price promotion, and placement). Models were validated using variance inflation factor (VIF) analysis for multicollinearity detection. All statistical analyses were performed using R Studio. The significance level was 5% for all analyses.

## RESULTS

**Figure 1:**
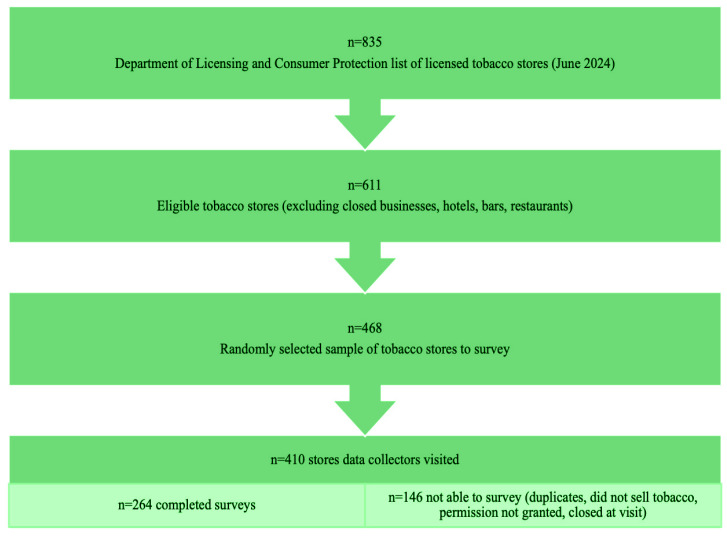
Cross-sectional study sampling strategy for tobacco retailers in the District of Columbia (DC) (N=264)

Of the 611 eligible stores, 468 stores were randomly selected to attempt to survey (Figure 1), assuming a 50% participation rate and a sample size objective of n=250. There was a 64% survey completion rate (n=264) of the 410 stores visited and surveyed. Surveys were not completed at some locations for the following reasons: permission to survey denied (n=40), store did not sell tobacco products (n=56), the business was no business operating at that location (n=15), the location was a duplicate entry (n=14), store was closed at time of visit (n=32), the environment was deemed unsafe (n=2), and the site was not attempted (n=45). Table 1 describes store characteristics, smoking prevalence, and socioeconomic status by DC geographical location (Ward). There were no significant differences in the proportion of registered tobacco licenses by Ward and the total stores surveyed by Ward.

**Table 1:** Smoking prevalence and poverty by District of Columbia (DC) Ward and sample tobacco retail store characteristics by Ward (N=264)

	*Ward*								
	*1*	*2*	*3*	*4*	*5*	*6*	*7*	*8*	*Average*
	*%*	*%*	*%*	*%*	*%*	*%*	*%*	*%*	*%*
**Smoking prevalence^a^**	20.9	21.1	22.7	29.9	27.9	24.8	27.9	44.7	25.8
**Poverty rate^b^**	11.6	11.2	7.4	8.7	14.7	11.3	22.4	26.8	14.5
	***Ward***								
	***1***	***2***	***3***	***4***	***5***	***6***	***7***	***8***	***Total***
	***n (%)***	***n (%)***	***n (%)***	***n (%)***	***n (%)***	***n (%)***	***n (%)***	***n (%)***	***n (%)***
**Registered tobacco license**	83 (13.5)	107 (17.5)	48 (7.9)	86 (14.1)	98 (16.0)	70 (11.5)	71 (11.6)	48 (7.9)	611 (100)
**Convenience store surveyed**	18 (10.9)	19 (11.5)	11 (6.7)	22 (13.3)	30 (18.2)	16 (9.7)	32 (19.4)	17 (10.3)	165 (62.5)
**Other store type surveyed**	13 (13.1)	17 (17.2)	10 (10.1)	16 (16.2)	13 (13.1)	15 (15.2)	9 (9.1)	6 (6.1)	99 (37.5)
**Stores not surveyed**	52 (6.7)	71 (20.5)	27 (7.8)	48 (13.8)	55 (15.9)	39 (11.2)	30 (8.6)	25 (7.2)	347 (100)
**Total stores surveyed**	31 (11.7)	36 (13.6)	21 (8.0)	38 (14.4)	43 (16.3)	31 (11.7)	41 (15.5)	23 (8.7)	264 (100)

a Lifetime smoking prevalence, 2023, ‘Have you smoked at least 100 cigarettes (5 packs) in your entire life?’ (https://dataviz1.dc.gov/t/OCTO/views/DCHEALTH-CPPE-BRFSS/RiskYesV2?%3AshowAppBanner=false&%3Adisplay_count=n&%3AshowVizHome=n&%3Aorigin=viz_share_link&%3Aembed=yes).

b Percent of District of Columbia residents living below the federal poverty level, 2024 (https://opdatahub.dc.gov/pages/district-of-columbia-profiles)

### Product availability

The majority of stores sold non-flavored cigarillos (80%, n=215), cigarettes (78%, n=206), and chew or loose tobacco (67%, n=176). Only 20% (n=52) of stores sold e-cigarettes. Convenience stores had the widest variety of tobacco products (both flavored and non-flavored). Pharmacies and grocery stores were the least likely to sell tobacco products. Availability of tobacco products differed by store type (convenience stores/all other) [χ^2^(2, N=264)=665.05, p<0.001] and Ward [χ^2^(7, N=264)=101.75, p<0.05]. Non-flavored cigarillos and non-flavored cigarettes were most frequently sold in convenience stores and liquor stores; non-flavored cigarillos and non-flavored cigarettes were also more frequently sold in Ward 7 and Ward 8 compared to all other Wards. The availability of menthol products did not differ by store type or Ward. Availability of flavored (non-menthol) products did not differ by Ward, but were more frequently available in convenience compared to other store types [χ^2^(2, N=264)=17.3, p<0.001]. Approximately 44% (n=115) of stores surveyed also sold alcoholic beverages. The majority of stores, 64% (n=168), displayed accurate tobacco-specific minimum age signs; however, only two stores (<1%) displayed graphic health warning signs about tobacco usage.

### Price

The average price for a pack of Marlboro Red cigarettes was $14.26 (range: 11–17.50, SD=1.05). The average price for a pack of Black and Mild cigarillos was $8.88 (range: 5.99–15.99, SD=2.01) and this differed by geography (F=5.18, p<0.001, 95% CI: 5.38–9.5) and store type (F=5.81, p<0.001, 95% CI: 8.43–9.34). Price was inversely associated with socioeconomic characteristics for cigarillos. Prices were higher, $10.94 on average (SD=2.80), in Ward 3, which has the highest average income compared to, $6.75 on average (SD=0.53), in Ward 8 which has the lowest average income. The overall mean price, $8.63 (SD=2.15), was significantly lower in convenience stores compared to other store types, with an average price of $9.24 (SD=1.58) (F=5.82, p<0.001, 95% CI: 8.22–10.93).

### Placement

The frequency of tobacco product placement within 12 inches of toys, candy, or gum differed significantly by Ward [χ^2^(7, N=264)=41.28, p<0.001] and store type [χ^2^(2, N=264)=16, p<0.001]. Wards with lower income communities were more likely to place products near these items than Wards within higher income communities. Convenience stores were significantly more likely than all other store types to have tobacco products placed within 12 inches of toys, candy, or gum [χ^2^(2, N=264)=16, p<0.001]. Convenience stores were also significantly more likely to display tobacco products within three feet of the floor [χ^2^(2, N=264)=8.43, p<0.05]. Non-convenience stores were more likely to have self-service tobacco products available [χ^2^(2, N=264)=16.45, p<0.001], specifically specialty tobacco stores and liquor stores frequently had self-service large cigars available.

### Promotion

Among tobacco products sold in DC, cigarillos most often had a price promotion, with 44% of cigarillos being offered with a price promotion. The frequency of price promotions on tobacco products differed significantly by Ward [χ^2^(7, N=264)=34.99, p<0.001] and store type [χ^2^(2, N=264)=13.4, p<0.01]. The percentage of stores with price promotions on tobacco products ranged from 14% of stores in highest income communities (Ward 3) to 78% of stores in lower income communities (Ward 8). Frequency of non-flavored tobacco products and menthol products advertising on exterior of stores ranged from 5% in the highest income community (Ward 3) to 67% in lower income communities (Ward 7), (Fisher’s exact, p<0.001). Convenience stores were significantly more likely to advertise non-flavored tobacco products [χ^2^(2, N=264)=24.9, p<0.001] non-menthol flavored products (Fisher’s exact, p<0.01), and menthol products (Fisher’s exact, p<0.01) outside of a store compared to all other store types. Only 35% (n=92) of all stores advertised tobacco products outside the retail environment.

Overall, convenience stores were more likely to have price promotions [χ^2^(2, N=264)=13.4, p<0.01] and non-flavored tobacco products advertised outside of a store [χ^2^(2, N=264)=24.9, p<0.001] compared to all other store types. Lower income communities (Wards 7 and 8) also had a higher frequency of price promotions [χ^2^(7, N=264)=34.99, p<0.001], tobacco products placed within 12 inches of toys, candy, or gum, [χ^2^(7, N=264)=41.28, p<0.001] and non-flavored tobacco products advertised outside of a store (Fisher’s exact, p<0.001).

### Regression models

Table 2 describes the final regression models, adjusting for confounding variables. The associations between the four Ps of marketing strategies (product, price, placement, and promotion) with geography and store types were similar to the bivariate analysis. There were consistent Ward-level geography effects across all models. In Model 1 (product availability) and Model 4 (placement of products), Ward 3, the highest income community, had the lowest product availability and placement near youth-oriented items relative to all other Wards. While availability was consistently higher across the other Wards, a few Wards (7, 5, and 2) stood out as having substantially greater product availability. Convenience stores were associated with increased tobacco product availability (β=8.51, SE=1.37; 95% CI: 5.82–11.2). In Model 2 pricing, all Wards predicted lower prices for Black and Mild cigarillos compared to Ward 3. In Model 3 promotions, significant geographical variation in the prevalence of price promotions was observed. Convenience stores were more likely to advertise price promotions (β=1.37, SE=0.34; 95% CI: 0.7–2.1).

**Table 2:** Multiple regression analysis for tobacco marketing strategies by geography and store type among tobacco retailers in the District of Columbia (DC) (N=264)

*Independent variable*	*Dependent variable*							
	*Model 1 Product availability (non-flavored)*		*Model 2 Price (Black & Mild)*		*Model 3 Price promotion*		*Model 4 Placement (near toys/candy)*	
	*Unadjusted β (SE) (95% CI)*	*Adjusted^a^ β (SE) (95% CI)*	*Unadjusted β (SE) (95% CI)*	*Adjusted^a^ β (SE) (95% CI)*	*Unadjusted β (SE) (95% CI)*	*Adjusted^a^ β (SE) (95% CI)*	*Unadjusted β (SE) (95% CI)*	*Adjusted^a^ β (SE) (95% CI)*
**Geography**								
Ward 3 ®								
Ward 1	3.97 (1.82) (0.41–7.53)*	2.71 (1.41) (0.15–5.47)*	2.09 (1.55) (-0.99–5.18)	1.88 (1.50) (1.1–4.86)*	1.70 (0.81) (1.1–3.3)*	1.59 (0.81) (0.09–3.45)*	1.68 (1.19) (–0.65–4.01)	1.60 (1.15) (0.7–3.85)*
Ward 2	6.41 (2.24) (2.02–10.8)*	5.54 (2.0) (1.62–9.4)*	3.15 (1.52) (0.12–6.18) *	2.86 (1.48) (0.08–5.81)*	0.1 (0.81) (-0.5–2.7)	1.09 (0.78) (0.45–2.63)*	1.79 (1.21) (–0.59–4.17)	1.54 (1.15) (0.71–3.79)*
Ward 4	5.97 (2.01) (2.7–10.7)*	4.71 (1.71) (1.36–8.1)*	2.30 (1.57) (-0.83–5.43)	2.02 (1.53) (1.03–5.07)*	1.68 (0.78) (1.44–3.22)*	1.5 (0.80) (0.03–3.24)*	2.72 (1.15) (0.47–4.97)*	2.55 (1.11) ( 0.37–4.73)*
Ward 5	6.71 (2.05) (2.7–10.7)*	5.82 (1.83) (2.23–9.41)*	-1.24 (1.67) (-4.55–2.07)	-1.34 (1.57) (-0.46–1.78)	2.56 (0.79) (-1.01–4.11)	2.31 (0.81) (0.82–4.0)*	3.20 (1.16) (0.92–5.48)*	2.85 (1.11) ( 0.67–5.1)*
Ward 6	6.44 (2.34) (1.86–11)*	5.38 (2.02) (1.42–9.34)*	1.54 (1.82) (-2.09–5.17)	1.23 (1.72) (1.19–4.65)*	0.27 (0.85) (-1.4–1.93)	0.18 (0.85) (-1.4–1.97)	1.59 (1.22) (–0.80–3.98)	1.25 (1.18) (1.07–3.57)*
Ward 7	7.22 (2.13) (3.04–11.4)*	6.28 (1.85) (2.66–9.9)*	1.18 (1.66) (-2.13–4.49)	1.08 (1.59) (0.08–4.24)*	2.77 (0.81) (1.17–4.4)*	2.48 (0.84) (0.95–4.28)*	3.24 (1.16) (0.96–5.52)*	2.94 (1.11) (0.75–5.12)*
Ward 8	5.21 (2.18) (0.93–9.49)*	4.18 (1.85) (1.55–7.81)*	-1.03 (1.74) (-4.49–2.43)	-1.23 (1.64) (-0.49–2.04)	3.45 (0.89) (1.7–5.2)*	3.09 (0.92) (1.39–5.05)*	3.92 (1.22) (1.53–6.31) *	3.44 (1.17) (1.14–5.74)*
**Store type**								
Other ®								
Convenience	1.2 (1.09) (0.94–3.34)*	1.51 (0.37) (1.12–2.2)*	-0.675 (0.77) (-2.18–0.83)	-1.16 (0.61) (0.39–0.07)*	1.37 (0.34) (0.7–2.1)*	1.17 (0.85) (1.4–1.97)*	1.41 (0.391) (0.64–2.18)*	1.49 (0.38) ( 0.75–2.23)*

a Adjusted for RICO-compliant signage –minimum age signage– and SNAP-participating stores. β: regression coefficient. SE: standard error. ® Reference categories.

* p<0.05.

## DISCUSSION

Tobacco marketing at the POS in retail environments remains one of the most influential strategies through which the tobacco industry promotes its products, especially as other advertising approaches have become increasingly regulated. The tobacco industry has consistently invested in POS marketing strategies, with an emphasis on retail price promotions^5,6,14^. These practices continue to pose challenges to public health initiatives aimed at reducing tobacco use. Our data demonstrate variation in POS tobacco marketing strategies by retail store type and geography.

Marketing strategies were found to be more frequent in convenience stores compared to all other store types. Marketing strategies were also more frequent in Wards with lower income communities compared to Wards with higher income communities. It is important to note that Ward 8 has the highest smoking prevalence and the highest percentage of residents living below the federal poverty level, while also having the lowest density of licensed tobacco retailers^20,22^. Convenience stores, which represent a large proportion of tobacco retailers in DC, were consistently associated with more frequent marketing strategies. Further, convenience stores and stores in Wards with lower income communities were significantly more likely to display tobacco products within 12 inches of candy, toys, or gum; offer price promotions on products such as cigarillos and menthol cigarettes; use outdoor advertising to promote non-flavored tobacco products; and exhibit a wider variety of tobacco products, including flavored varieties. Of note, POS marketing strategies of cigarillos were disproportionately available, marketed, and lower priced in lower income communities compared to higher income communities^27^. Although liquor stores sold a limited range of products, they typically sold non-flavored cigarillos and cigarettes. Pharmacies and grocery stores were the least likely to sell tobacco products.

These findings are consistent with broader national patterns in tobacco POS marketing^5,28^. Rose et al.^6^ and Kong et al.^13^ found that the density of flavored tobacco product marketing was highest in low-income neighborhoods with a high proportion of Black residents. Consistent with our findings, Rose et al.^6^ also found that convenience stores were more likely to employ a variety of tobacco marketing strategies. These results underscore the need for comprehensive national and state tobacco control strategies that address the variability of tobacco product promotion at POS by Ward and within retail environments.

Additional studies are necessary to examine the causal associations between exposure to marketing strategies and smoking prevalence, and the effectiveness of policy strategies to decrease use of tobacco products^29^. In DC, regulations on tobacco access focus on restriction of tobacco product sales to minors under 21 years of age, bans on flavored tobacco products and, restrictions on tobacco product sales in vending machines or self-service displays (DC law does allow self-service displays for large cigars in tobacco stores)^24,25^. DC also restricts the display of tobacco products and the placement of tobacco products adjacent to candy, toys, or other products near children, and youth^24^. However, there are currently no restrictions on the number of tobacco licenses in DC^25^. A separate license is required for each retail store selling tobacco products that must be renewed annually^25^, which provides an opportunity to limit the number of tobacco licenses across DC. The 2009 Tobacco Control Act grants the Food and Drug Administration (FDA) the authority to regulate tobacco products, including their placement and promotion in retail outlets, and in 2016, extended this authority to e-cigarettes, cigars, hookah, pipe tobacco, and nicotine gels^16^. However, the federal government does not have the authority to regulate tobacco retailer licensing and density in a jurisdiction. State and local governments may use their authority to regulate and enforce restrictions on the tobacco retail environment^16^.

In addition to marketing practices, pricing remains one of the most effective tools for reducing tobacco use^30^. However, existing pricing state policies often only regulate cigarettes, not cigarillos and other tobacco products. State law that standardizes excise taxes on all tobacco products would support price parity across product types. Additional state-level recommendations include: 1) requiring tobacco products be kept out of view in the retail environment; 2) prohibiting advertising in retail stores near cash registers; 3) requiring and enforcing health warning display signs; and 4) limiting the number of tobacco retail outlets, including proximity to areas with high density of youth^31,32^.

### Limitations

This study has limitations. Although the tobacco retail stores were randomly selected and had a high participation rate, there may have been selection bias given the stores that agreed to participate and were open between 10 a.m. and 3 p.m., according to the study protocol, may not be representative of all tobacco retailers in DC. Marketing strategies were assessed as dichotomous (yes or no) responses which limited the ability to specify the quantity and types of advertising approaches by store type and location. Data collectors recorded graphic warning health signs about tobacco usage; however, accurate information was not available to determine which tobacco retailers were mandated to display corrective statements on the risks of smoking tobacco products according to the 2023 determination to support the Racketeer Influenced and Corrupt Organizations Act (RICO), purposeful violation by tobacco companies regarding the risks of smoking. Causal relationships are not possible to determine given the cross-sectional study design. It is important to consider the impact of residual confounding when interpreting the associations in this study due to confounders not accounted for. The generalizability of these findings is limited due to the restriction of the study sample to DC.

## CONCLUSIONS

This study demonstrates variation in POS tobacco marketing strategies by retail store type and geography. Marketing strategies were more frequent in convenience stores compared to all other store types. Marketing strategies were also more frequent in Wards with lower income communities compared to Wards with higher income communities. Additional research on feasibility and effectiveness of point-of-sale marketing practices of tobacco products will guide regulatory interventions and policies.

## Data Availability

The data supporting this research are available from the authors on reasonable request.
